# Ferroptosis in Gastrointestinal Diseases: A New Frontier in Pathogenesis and Therapy

**DOI:** 10.3390/jcm14124035

**Published:** 2025-06-07

**Authors:** Adam Wawrzeńczyk, Katarzyna Napiórkowska-Baran, Ewa Alska, Alicja Gruszka-Koselska, Ewa Szynkiewicz, Józef Sławatycki, Paula Klemenska, Zbigniew Bartuzi

**Affiliations:** 1 Department of Allergology, Clinical Immunology and Internal Diseases, Collegium Medicum Bydgoszcz, Nicolaus Copernicus University in Torun, 85-067 Bydgoszcz, Poland; adam.wawrzenczyk@cm.umk (A.W.); ewa.alska@cm.umk.pl (E.A.); alicja.gruszka@op.pl (A.G.-K.); zbartuzi@cm.umk.pl (Z.B.); 2 Department of Nursing in Internal Diseases, Collegium Medicum Bydgoszcz, Nicolaus Copernicus University Torun, 85-067 Bydgoszcz, Poland; ewaszynkiewicz@wp.pl; 3 Jan Biziel University Hospital No. 2, 85-168 Bydgoszcz, Poland; jozef.slawatycki@cm.umk.pl (J.S.); paula.klemenska@biziel.pl (P.K.); 4 Department of Pharmacology and Therapeutics, Faculty of Medicine, Collegium Medicum in Bydgoszcz, Nicolaus Copernicus University in Torun, M. Curie Skłodowskiej 9, 85-094 Bydgoszcz, Poland

**Keywords:** ferroptosis, gastrointestinal diseases, iron metabolism, oxidative stress, inflammatory bowel disease, liver disorders, colorectal cancer, GPX4, lipid peroxidation, targeted therapy

## Abstract

Ferroptosis, a form of regulated cell death driven by iron-dependent lipid peroxidation, has emerged as a key player in the pathogenesis of gastrointestinal (GI) diseases. Unlike apoptosis or necrosis, ferroptosis is characterized by distinctive metabolic and molecular pathways, including dysregulated iron metabolism, oxidative stress, and impaired antioxidant defenses. This review explores the complex role of ferroptosis in conditions such as inflammatory bowel disease (IBD), non-alcoholic steatohepatitis (NASH), and gastrointestinal cancers. Special attention is given to the molecular mechanisms underlying ferroptosis, including the Xc^−^/GSH/GPX4 axis, ferritinophagy, ACSL4/LPCAT3-mediated lipid remodeling, and the influence of the gut microbiota. Therapeutic strategies targeting ferroptosis—including pharmacological inhibitors, iron chelators, and microbiota-based interventions—are evaluated for their translational potential, underscoring ferroptosis as a promising target for precision therapies in gastroenterology and highlighting the need for further clinical studies to validate its diagnostic and therapeutic implications.

## 1. Introduction

The human body, a complex network of cellular and molecular interactions, relies on the precise regulation of cell death to maintain tissue homeostasis and respond to pathological insults [1]. Among the diverse forms of regulated cell death, ferroptosis, an iron-dependent mechanism, has emerged as a distinct form characterized by lipid peroxidation and oxidative stress. Unlike apoptosis or necrosis, ferroptosis exhibits unique molecular features that underscore its dual role in physiological and pathological contexts [2]. Recent research highlights its involvement in various diseases, including gastrointestinal disorders, which are associated with high oxidative stress and iron abundance. Understanding the implications of ferroptosis in these diseases opens new possibilities for therapeutic strategies [3].

Gastrointestinal diseases, encompassing conditions such as colorectal cancer, gastric cancer, inflammatory bowel diseases (IBD), and chronic liver disorders, represent a substantial global health burden. The discovery of ferroptosis as a regulated cell death pathway has been pivotal in advancing our understanding of these disorders, particularly due to its relevance in iron metabolism and lipid peroxidation [4]. Emerging evidence suggests that ferroptosis contributes to the pathogenesis of these diseases and, conversely, holds potential as a therapeutic target [5]. However, several gaps in knowledge persist, particularly regarding biomarkers, the adverse effects of therapeutic interventions, and the precise mechanisms linking ferroptosis to gastrointestinal pathology [6].

This paper aims to elucidate the molecular mechanisms of ferroptosis and its regulatory systems, focusing on key components such as iron metabolism, lipid peroxidation, and the Xc^−^/GSH/GPX4 axis—a cellular antioxidant pathway that protects cells from ferroptosis by importing cystine (via system Xc^−^), synthesizing glutathione (GSH), and utilizing glutathione peroxidase 4 (GPX4) [7]. It investigates the connection between ferroptosis and gastrointestinal diseases, shedding light on its contribution to conditions such as IBD, liver disorders, and gastrointestinal cancers.

**Figure 1:**
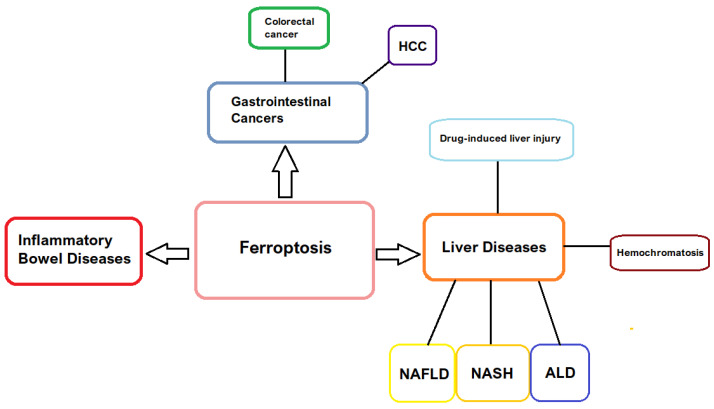
Summary of ferroptosis-associated gastrointestinal diseases. Definitions of abbreviations: HCC—hepatocellular carcinoma; NAFLD—non-alcoholic fatty liver disease; NASH—non-alcoholic steatohepatitis; ALD—alcoholic liver disease.

Furthermore, the study evaluates the therapeutic potential of modulating ferroptosis through both inhibitors and inducers, considering the dual nature of ferroptosis in disease progression and therapy. Figure 1 shows a summary of the gastrointestinal diseases associated with ferroptosis discussed in this manuscript.

To guide the reader through this review, Section 2 focuses on the molecular mechanisms underpinning ferroptosis, including its regulation by iron metabolism, oxidative stress, and essential enzymes like GPX4. Section 3 explores the role of ferroptosis in gastrointestinal pathology, specifically its impact on inflammatory bowel diseases and liver disorders. Section 4 and Section 5 discuss therapeutic approaches targeting ferroptosis, including emerging strategies for drug development, microbiota interactions, and the synergy between ferroptosis modulation and standard treatments, while also identifying critical areas for future research.

## 2. Molecular Mechanism of Ferroptosis

Ferroptosis has emerged as a critical cell death mechanism characterized by iron-dependent lipid peroxidation, profoundly impacting cellular homeostasis and health. The intricate interplay between iron metabolism, oxidative stress, and key regulatory systems underpins the pathophysiology of this unique form of cell death [8].

### 2.1. Iron Metabolism and Oxidative Stress

**Figure 2:**
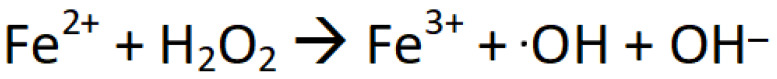
Fenton reaction.

Iron metabolism and oxidative stress are the main regulators of ferroptosis, a form of regulated cell death driven by lipid peroxidation. Excess iron accumulates in the labile iron pool, where its mismanagement can trigger the Fenton reaction—a process in which iron catalyzes the conversion of hydrogen peroxide into highly reactive hydroxyl radicals [9]. Figure 2 illustrates the Fenton reaction.

These radicals initiate lipid peroxidation, damaging polyunsaturated fatty acids (PUFAs) within cellular membranes and disrupting their structural integrity, ultimately leading to ferroptotic cell death [7].

Byproducts of lipid peroxidation, such as malondialdehyde (MDA), not only exert cytotoxic effects but also serve as quantifiable biomarkers for ferroptosis, aiding in the assessment of cell death in various pathological contexts [10]. The physical disruption of lipid bilayers caused by oxidative damage to membranes results in ion imbalances, ultimately triggering cell death. This destabilization poses particular challenges for tissues with high turnover rates such as the gastrointestinal epithelium [11]. Moreover, lipid peroxidation not only drives ferroptotic death but also amplifies inflammatory signals, further increasing oxidative stress [11].

Lipid peroxidation leads to the formation of reactive products such as MDA and 4-hydroxynonenal (4-HNE), which can interact with DNA, forming DNA adducts that initiate apoptotic cascades, including the activation of DNA damage response (DDR) pathways [12]. The DDR pathway is a highly conserved cellular mechanism activated by diverse forms of DNA damage to halt cell proliferation, facilitate DNA repair processes, and eliminate irreparably damaged cells, thereby preserving genomic stability [12]. Xu et al. (2021) demonstrated that MDA and 4-HNE interact with DNA, activating ATR and ATM kinases, which promote apoptosis by initiating signaling pathways involving p53 [13]. Chen et al. (2021) emphasize that lipid peroxidation induces mitochondrial membrane alterations, leading to cytochrome c release and the activation of caspase cascades, confirming the pivotal role of oxidative stress in the initiation of apoptosis in colorectal cancer cells [14]. Additionally, Zeng et al. (2023) highlight that lipid peroxidation products can serve as biomarkers of DNA damage and may be utilized to monitor the extent of oxidative stress and disease progression in gastrointestinal disorders [15].

Zhao et al. [16] investigated in vivo the impact of iron overload on the proliferation and differentiation of intestinal stem cells in mice. Mice were administered 120 mg/kg of iron dextran intraperitoneally every 14 days for 12 weeks. The study revealed that chronic iron excess severely damaged intestinal epithelial cells, reduced stem cell proliferation, and altered cellular morphology. These findings provide critical insights into how iron dysregulation may serve as both a cause and a consequence of gastrointestinal pathologies.

Oxidative stress arises from an imbalance between reactive oxygen species (ROS) production and cells’ antioxidant capacity, playing a central role in driving ferroptosis [17]. ROS preferentially target the PUFAs found in cellular membranes, initiating chain reactions of lipid peroxidation [17]. These chain reactions culminate in membrane rupture and ferroptotic death, highlighting the destructive potential of excessive ROS levels. Such elevated ROS levels further induce oxidative damage to essential cellular components like proteins and DNA, compounding overall tissue injury [18]. Tang et al. [19] highlight the critical relationship between oxidative stress and the damage wrought by ferroptosis, particularly within disease contexts where ROS production overwhelms antioxidant defenses. This relationship links excess iron to cascading effects, including membrane destabilization and increased cellular injury. This damage is particularly pronounced in the gastrointestinal tract, where it exacerbates inflammation and amplifies tissue degeneration, creating a vicious cycle of ferroptosis and oxidative stress [19].

### 2.2. Regulatory Systems

The dual role of iron as both an essential nutrient and a trigger for ferroptosis highlights the importance of precisely regulating iron-related pathways. Achieving a balance between ensuring sufficient iron for critical metabolic functions and limiting its excess to prevent ROS-driven damage is a key priority for maintaining gastrointestinal health [20].

Iron metabolism is tightly regulated by key molecular mediators, including hepcidin, a liver-derived hormone that maintains systemic iron homeostasis. Hepcidin is responsible for regulating irons levels in the human body by inhibiting absorption from the gastrointestinal tract and iron release from the reticuloendothelial system [14]. The dysregulation of hepcidin has been strongly associated with heightened sensitivity to ferroptosis due to its role in enabling iron overload [21].

Nuclear factor erythroid 2–related factor 2 (Nrf2) is a master regulator of antioxidant defense and plays a multifaceted role in ferroptosis. By promoting the transcription of detoxifying enzymes and stress-response genes, Nrf2 enhances cellular resistance to oxidative stress and ferroptosis [22]. Conversely, Nrf2 suppression permits unchecked lipid peroxide accumulation, intensifying ferroptotic damage. Cheng et al. [23] demonstrated that the inactivation of Nrf2 diminishes antioxidant capacity, further amplifying susceptibility to ROS-induced damage and ferroptosis. These findings underscore the intricate regulatory networks governing the delicate interplay between iron metabolism and oxidative stress pathways. In gastrointestinal diseases such as IBD and liver disorders, dysregulated Nrf2 signaling has been linked to heightened ferroptosis sensitivity. Experimental data suggest that activating Nrf2 in these contexts can alleviate tissue damage and inflammation, underscoring its therapeutic relevance [24].

Glutathione peroxidase 4 (GPX4) is an antioxidant enzyme responsible for detoxifying lipid peroxides by reducing toxic lipid hydroperoxides into harmless alcohols using reduced glutathione (GSH) as a cofactor [25]. The interplay between iron homeostasis and the enzymatic activity of GPX4 emerges as a pivotal factor in ferroptosis regulation [25,26]. It protects cells from the accumulation of reactive lipid species and the intensification of ferroptotic cell injury [25]. However, its activity is severely compromised under conditions of iron overload or glutathione depletion, enabling ferroptosis to proceed unchecked [26]. This dynamic has been explored in IBD, wherein the inhibition of GPX4 exacerbates intestinal damage [3]. These insights into the GPX4-dependent mechanisms of ferroptosis open pathways for therapeutic strategies aimed at mitigating oxidative damage [3].

**Figure 3:**
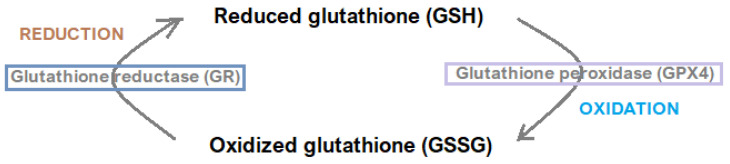
Glutathione redox cycle.

The Xc^−^/GSH/GPX4 axis represents a central regulatory mechanism in ferroptosis, with its functionality determining cells’ ability to counteract oxidative stress [27]. System Xc^−^ is a cystine/glutamine antiporter composed of two subunits: SLC7A11 and SLC3A2. It imports extracellular cystine into the cell in exchange for intracellular 1-glutamate [27,28]. Cystine is subsequently converted intracellularly into cysteine—a precursor essential for GSH synthesis in the cysteine/GSH/GPX4 pathway [27,28]. Glutathione is a tripeptide composed of cysteine, glutamate, and glycine. It occurs in two main forms: a glutathione disulfide (GSSG)-oxidized form and reduced glutathione (GSH) [27,28,29]. Figure 3 illustrates the glutathione redox cycle.

As a critical cofactor, GSH directly supports glutathione peroxidase 4 (GPX4) in neutralizing lipid peroxides, thereby halting the progression of ferroptosis [7,27]. However, disruptions in this axis—such as GPX4 inactivation caused by RAS-selective lethal 3 (RSL3)—lead to the accumulation of harmful lipid peroxidation products and ferroptotic cell death [25]. This mechanistic insight underscores the axis’s vital role in maintaining redox equilibrium [18]. Research into this axis has revealed its dual therapeutic significance: inhibition can serve as a strategy to induce ferroptosis in cancer cells, while enhancement may protect against ferroptosis-induced damage in non-malignant diseases. These findings highlight the necessity for precision medicine approaches that consider the pathological context before modulating this pathway [7,18,27].

The enzymes acyl-CoA synthetase long-chain family member 4 (ACSL4) and lysophosphatidylcholine acyltransferase 3 (LPCAT3) are indispensable in lipid remodeling processes that culminate in ferroptosis [22,30]. These enzymes mediate the incorporation of polyunsaturated fatty acids (PUFAs), such as arachidonic acid (AA) and adrenic acid (AdA), into phospholipids—preferred substrates for lipid peroxidation. Experimental data indicate that the suppression or deletion of ACSL4 or LPCAT3 significantly reduces ferroptosis, reaffirming their central role in this cell death pathway [22]. This mechanism is particularly relevant in gastrointestinal tissues, where oxidative stress readily promotes lipid peroxidation. For instance, ACSL4 expression is markedly elevated in inflamed intestinal tissues, correlating with ferroptotic damage in conditions like inflammatory bowel disease [3].

**Figure 4:**
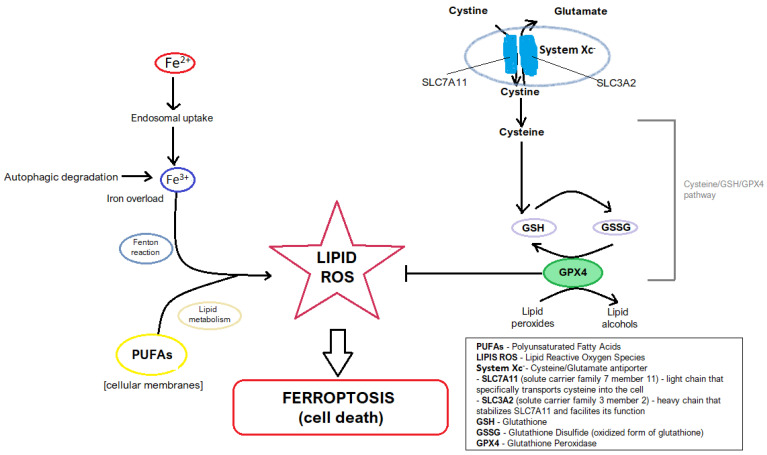
The general mechanism of ferroptosis.

Beclin1 (BECN) is a protein that regulates the imitation of autophagy [18,31]. The interaction between Beclin 1 (BECN1) and SLC7A11—one of two subunits of the Xc^−^ system—adds another regulatory dimension to ferroptosis by modulating cystine uptake and intracellular redox balance. Beclin 1 inhibits SLC7A11 activity, reducing cystine transport and consequently depleting intracellular glutathione levels, which heightens susceptibility to ferroptosis [18]. This interaction is particularly relevant in colorectal cancer subtypes, where it influences tumor progression and therapy resistance. Understanding the functional dynamics between these proteins opens therapeutic possibilities—specifically, selectively triggering ferroptosis in cancer cells while sparing normal tissue [32]. Figure 4 shows the general mechanism of ferroptosis.

### 2.3. Ferritinophagy

**Figure 5:**
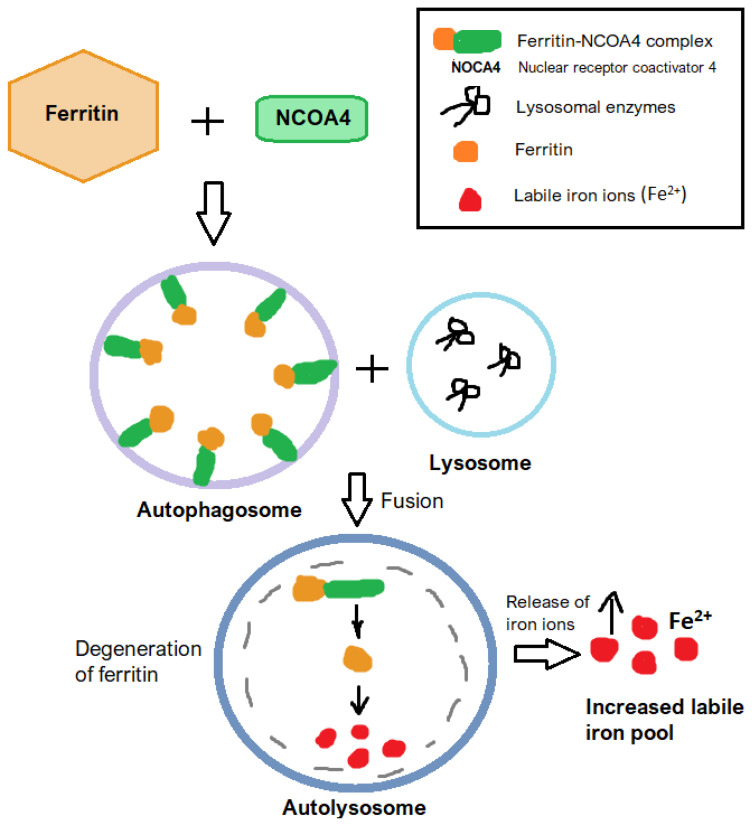
The general mechanism of ferritinophagy.

Autophagy, especially the selective process of ferritinophagy, represents another key regulatory mechanism of ferroptosis. In ferritinophagy, ferritin—the primary intracellular iron storage complex—is degraded in lysosomes, leading to an increased labile iron pool [33]. This process is mediated by nuclear receptor coactivator 4 (NCOA4) which binds ferritin and delivers it to the autophagosome [34,35]. Elevated iron availability amplifies lipid peroxidation and drives ferroptosis [14]. The activation of autophagy and ferritinophagy pathways has been implicated in gastrointestinal pathologies, where it contributes to oxidative stress and lipid ROS accumulation [34]. This pro-ferroptotic role is particularly evident in diseases like IBD andNAFLD, where it exacerbates tissue injury and disease progression [36]. Figure 5 shows the general mechanism of ferritinophagy.

## 3. Ferroptosis in Gastrointestinal Pathology

### 3.1. Inflammatory Bowel Disease

Inflammatory Bowel Disease (IBD) is a term for a group of non-specific chronic inflammatory disorders of the gastrointestinal (GI) tract [37]. There are two main medical subtypes of this condition—ulcerative colitis (UC) and Crohn’s disease (CD). UC primarily affects the colon and rectum, causing inflammation and ulceration of the epithelial layer, and, to a lesser degree, the submucosal layer of the larger intestine. However, CD can affect any part of the digestive system from the mouth to the anus, involving all layers of the intestine wall [14]. Ferroptosis plays a pivotal role in the development and progression of IBD, primarily through mechanisms involving iron accumulation, lipid peroxidation, and subsequent epithelial cell death and chronic inflammation. Elevated markers of lipid peroxidation, such as MDA, along with increased intracellular iron levels and depleted antioxidant components like GSH and GPX4, have been consistently observed in IBD patients [38]. This biochemical profile positions ferroptosis as a central contributor to IBD pathology. The heightened MDA levels reflect extensive oxidative damage, reinforcing the link between lipid peroxidation and epithelial injury [3,38]. Iron accumulation in inflamed tissues promotes ROS generation via the Fenton reaction, intensifying oxidative stress and perpetuating ferroptosis-mediated intestinal damage, thus creating a self-reinforcing inflammatory cycle [39].

The disruption of the cysteine/GSH/GPX4 antioxidant system due to GSH depletion and GPX4 inactivation further exacerbates oxidative stress, promoting lipid peroxidation and ferroptotic epithelial cell death [40]. GSH levels are significantly reduced in IBD due to impaired biosynthesis, limited cysteine availability, and excessive oxidative stress that depletes GSH in response to elevated ROS [19,40]. This disruption weakens antioxidant defense, exacerbates lipid peroxidation, and promotes ferroptosis and epithelial damage. This process leads to compromised barrier integrity and sustained inflammation, highlighting the therapeutic relevance of restoring redox homeostasis [5]. Iron overload amplifies these effects, underscoring the need for interventions targeting both iron metabolism and antioxidant defense to interrupt the feedback loop of ferroptotic damage [5,40].

Key regulatory pathways, including the cysteine/GSH axis and acyl-CoA synthetase long-chain family member 4 (ACSL4), are crucial to ferroptotic mechanisms in IBD. Inflammatory stimuli upregulate ACSL4 expression in intestinal tissues, particularly in Crohn’s disease and ulcerative colitis, enhancing PUFA-containing phospholipid synthesis and increasing susceptibility to lipid peroxidation [38,40]. Targeting ACSL4, via siRNA knockdown or pharmacological inhibitors like rosiglitazone, has demonstrated efficacy in reducing inflammation and promoting mucosal healing in preclinical models. While promising, this strategy requires careful modulation to avoid unintended metabolic disruptions [38].

Ferroptosis-induced intestinal barrier dysfunction is further mediated by the mechanosensitive ion channel Piezo1, whose overexpression seems to be genetically determined and it is elevated among patients suffering from IBD [40]. Its overexpression impairs tight junction integrity and exacerbates inflammation. In animal models, Piezo1 deletion mitigates ferroptosis and restores barrier function via regulation of the AMPK/mTOR signaling pathway. Wang et al. [41] observed in vivo, in mice suffering from IBD, that the absence of piezo1 in macrophages was associated with reduced intestinal inflammation. These findings suggest that Piezo1 is a potential therapeutic target for reducing epithelial injury in IBD. Piezo1-specific inhibitors may enhance barrier stability and mitochondrial function, though their systemic effects warrant careful evaluation due to Piezo1’s broader physiological roles [42].

The gut microbiota also modulates ferroptosis in IBD. Pathogenic bacteria such as Fusobacterium nucleatum induce ferroptosis by increasing Fe^2+^ accumulation and lipid peroxidation, leading to epithelial barrier compromise. Infected tissues show elevated MDA and oxidative stress markers, implicating microbial activity in accelerating ferroptotic damage [43]. Ferroptosis inhibitors, including Ferrostatin-1, alleviate barrier damage and inflammation in these contexts, highlighting the therapeutic potential of microbiota-targeted ferroptosis modulation. Nonetheless, further research is needed to delineate the precise mechanisms of microbial-induced ferroptosis and its broader impact on gut homeostasis [43].

Genetic and molecular alterations, such as reduced expression of the E3 ubiquitin ligase NEDD4L, further implicate ferroptosis in IBD. NEDD4L deficiency exacerbates colitis by intensifying oxidative stress and lipid peroxidation. Conversely, restoring NEDD4L expression reduces epithelial injury and inflammation, suggesting a protective regulatory role [44]. Therapeutic strategies focused on enhancing NEDD4L activity or directly inhibiting ferroptosis offer promising avenues for personalized treatment approaches. Integrating NEDD4L-based biomarkers into diagnostic frameworks may improve patient stratification and therapeutic precision [44].

The pharmacological modulation of ferroptosis has demonstrated therapeutic promise in IBD. Agents such as metformin, which activate the AMPK pathway, suppress ferroptosis by reducing lipid peroxidation and enhancing antioxidant responses. Similarly, regulatory molecules like GZMA have shown protective effects against ferroptotic epithelial injury and inflammation [45]. Combination therapies employing ferroptosis inhibitors and metabolic activators present an opportunity to address the multifaceted nature of IBD. Translating these preclinical findings into clinical practice will require further investigation into safety, efficacy, and optimal dosing strategies [46].

### 3.2. Liver Disorders 

Ferroptosis is a pivotal pathological mechanism underlying hepatocyte injury in liver disorders, driven primarily by lipid peroxidation and oxidative stress [4,15]. The excessive accumulation of lipid peroxides initiates inflammatory responses, hepatocellular ballooning, and fibrosis [4,15]. Iron deposition exacerbates these processes by intensifying ROS generation, thereby amplifying cellular damage. This dual role—both initiating and perpetuating liver injury—positions ferroptosis as a central element in the pathogenesis of hepatic disorders [4,15].

Non-alcoholic fatty liver disease (NAFLD) encompasses a spectrum of liver disorders characterized by excessive fat accumulation in the liver (hepatic steatosis) among non-alcohol abusers. NAFLD is closely associated with metabolic risk factors such as obesity, insulin resistance, type 2 diabetes, and dyslipidemia [47]. It ranges from simple steatosis (non-alcoholic fatty liver, or NAFL) to non-alcoholic steatohepatitis (NASH) [36]. NASH is a progressive form of NAFLD characterized by hepatic steatosis, inflammation, and hepatocellular injury, with or without fibrosis. It can progress to cirrhosis, liver failure, and hepatocellular carcinoma (HCC) [48]. The progression from NAFLD to NASH is closely linked to ferroptotic mechanisms. Oxidative stress and iron overload promote lipid peroxidation and ferroptosis-induced hepatocyte death, accelerating fibrosis and liver dysfunction [48]. Therapeutically targeting these pathways may offer a promising strategy for halting disease advancement, although pinpointing specific molecular targets remains challenging due to the multifactorial nature of NAFLD [15,49].

Lipid peroxidation, a hallmark of ferroptosis, is particularly detrimental in the liver due to its central role in lipid metabolism and vulnerability to oxidative stress. The metabolic activity of hepatocytes creates conditions conducive to ferroptotic injury, reinforcing lipid peroxidation’s role in liver damage and fibrotic remodeling in disorders like NASH [4,49]. Marta Martín-Fernández et al. reported that higher lipid peroxidation levels are independently associated with a greater risk of developing NASH. Researchers revealed that control of lipid peroxidation level is a valuable diagnostic tool for identifying NASH patients among NAFLD sufferers [49]. By focusing on the suppression of lipid peroxidation and oxidative stress, therapeutic strategies could directly target the pathogenic mechanisms fueling disease progression [50]. This highlights the therapeutic potential of ferroptosis modulation in addressing hepatocyte injury and liver dysfunction, particularly in advanced stages of NAFLD [49,50].

Iron overload is another major contributor to ferroptosis in hepatic pathologies, including alcoholic liver disease (ALD) and hereditary hemochromatosis [51]. Excess iron catalyzes ROS generation via the Fenton reaction, triggering lipid peroxidation and hepatocyte death [4,51]. Managing iron dysregulation is therefore essential for preventing ferroptosis-mediated liver injury [15]. In ALD, increased serum ferritin levels and dysregulated iron transport intensify oxidative stress and ferroptosis [52]. This imbalance worsens liver inflammation and fibrosis, emphasizing the pathological relevance of iron metabolism. Therapies aimed at reducing iron accumulation, such as chelators or dietary modifications, show promise in mitigating liver damage [34,51]. Pathological iron accumulation may also serve as a diagnostic indicator of disease severity [4,51,52]. The upregulation of divalent metal transporters in ALD, for instance, facilitates hepatic iron overload and confirms the centrality of ferroptosis in disease progression. These insights highlight the potential of targeted strategies for iron homeostasis regulation [52]. Preclinical studies have demonstrated the effectiveness of ferroptosis-targeting agents. Ferrostatin-1, a ferroptosis inhibitor, protects against hepatocyte injury and fibrosis in ALD and NASH [51]. Ferrostatin-1 prevents lipid peroxidation by neutralizing lipid reactive oxygen species and stabilizes the cell membrane [53,54]. Similarly, iron chelators such as deferoxamine reduce ROS production and ferroptosis-related damage in iron-overload conditions [13]. Nevertheless, further studies are needed to assess their long-term safety and clinical viability [24].

The Nrf2 signaling pathway plays a protective role by upregulating antioxidant systems, notably GPX4 [19]. Pharmacological agents such as nobiletin activate Nrf2 and attenuate ferroptosis, reducing liver injury in models of sepsis-associated liver damage [55]. Conversely, diminished Nrf2 activity heightens susceptibility to ferroptosis by impairing antioxidant responses, as shown in NAFLD and ALD models. Therapeutic enhancement of Nrf2 or its downstream targets, like GPX4, offers a promising approach for protecting hepatocytes from oxidative damage [4,15,52]. While Nrf2 activation offers therapeutic benefits, its modulation requires careful calibration to avoid adverse outcomes linked to overactivation. This dual role highlights the need for personalized therapeutic strategies [52,55].

In HCC, ferroptosis exerts a paradoxical effect—acting both as a tumor suppressor and a mediator of treatment resistance [56]. While its induction may promote selective tumor cell death, adaptive responses can undermine therapies like sorafenib by counteracting oxidative stress [49,56]. This complexity necessitates the precise modulation of ferroptosis to achieve therapeutic efficacy without fostering resistance [13,56].

Autophagy, particularly ferritinophagy—the selective degradation of ferritin—also contributes to ferroptosis by increasing intracellular iron levels [34]. This promotes lipid peroxidation and hepatocellular injury, particularly in chronic liver diseases. Dysregulated autophagy exacerbates ferroptotic damage, reinforcing its pathogenic significance [57]. The targeted inhibition of ferritinophagy, while preserving essential autophagic functions, may represent a viable therapeutic avenue. Modulating specific regulatory proteins in this process could help limit ferroptosis without disrupting overall cellular homeostasis [15,57].

## 4. Therapeutic Targets of Ferroptosis

Ferroptosis, a regulated form of cell death driven by iron-dependent lipid peroxidation, has been increasingly recognized as a critical mechanism in the pathogenesis of various gastrointestinal and inflammatory diseases [23,57]. Recent studies have identified several promising therapeutic strategies to modulate ferroptosis, including the regulation of iron metabolism, enhancement of antioxidant defenses, and inhibition of lipid peroxidation, and the application of iron chelators to reduce labile iron pools and mitigate oxidative damage [57]. Notably, ferritinophagy—a selective autophagic process that degrades ferritin to release free iron—has emerged as a pivotal upstream regulator of ferroptosis, linking autophagy to iron homeostasis and cellular susceptibility [35,57]. Pharmacological interventions targeting the Nrf2 pathway, the Xc^−^/GSH/GPX4 axis, ferritinophagy, and lipid remodeling enzymes have demonstrated encouraging effects in preclinical models [57]. However, the translation of these findings into safe and effective clinical applications remains challenging, requiring a more comprehensive understanding of tissue-specific vulnerabilities and potential off-target effects [57].

Pharmacological agents that modulate Nrf2 activity have shown promise in treating ferroptosis-related disorders. Compounds like nobiletin, which upregulate Nrf2, have demonstrated efficacy in enhancing antioxidant defenses and mitigating ferroptosis [55]. While these results are encouraging, the clinical viability of Nrf2-targeted therapies requires deeper evaluation, particularly regarding their safety profiles and the risk of disrupting cellular homeostasis through Nrf2 overactivation [4].

Excessive iron accumulation, often observed in hereditary hemochromatosis or chronic inflammatory conditions like IBD, promotes oxidative stress and lipid peroxidation, increasing cellular vulnerability to ferroptosis [5,58]. Iron chelators such as deferoxamine have demonstrated protective effects against ferroptosis-related injury in preclinical models, reinforcing the therapeutic potential of strategies aimed at regulating iron homeostasis [3,14]. Deferoxamine inhibits ferroptosis by reducing intracellular iron levels, thereby limiting lipid peroxidation—a key driver of this form of cell death [59].

Therapeutic measures designed to counter oxidative stress have shown potential, including antioxidant compounds that neutralize ROS and agents that restore the cysteine/GSH/GPX4 pathway. These targeted strategies are effective in protecting epithelial cells from ferroptotic damage [3,5].

Therapeutics that inhibit lipid peroxidation, such as Ferrostatin-1, have shown initial promise in preclinical studies. These treatments block lipid peroxidation, stabilize cellular membranes, and reduce ROS-induced damage, offering potential relief in conditions like IBD and NAFLD, where ferroptosis is implicated as a pathogenic mechanism [53].

The therapeutic effectiveness of targeting the Xc^−^/GSH/GPX4 axis depends on tissue-specific vulnerabilities. Gastrointestinal tissues, exposed to high levels of oxidative byproducts due to their metabolic activity, are particularly susceptible to disruptions in this system [5]. Pre-existing conditions such as iron overload exacerbate this vulnerability, as excess iron promotes oxidative stress and lipid peroxidation, further impairing the axis [57]. Studies suggest that oxidative stress disproportionately impacts epithelial cells in the gastrointestinal tract, leading to barrier dysfunction and inflammation, thus linking this axis to gastrointestinal pathologies [58]. Effective therapeutic interventions targeting this axis therefore require comprehensive analyses of tissue-specific factors to optimize treatment strategies [57].

The pharmacological inhibition of ACSL4 and LPCAT3 has emerged as a promising therapeutic approach. The genetic silencing or chemical inhibition of ACSL4 has shown efficacy in reducing ferroptosis in preclinical models of cancer and inflammatory diseases [22,24]. These findings provide a foundation for therapies aimed at blocking lipid remodeling processes that are crucial for ferroptosis. However, the potential for off-target effects and tissue-specific consequences remains an open question, requiring further investigation [22,24].

From a therapeutic standpoint, targeting ferritinophagy offers a promising avenue for mitigating ferroptosis-induced damage [34]. Fang et al. [60] observed that ferroptosis inhibitor 9a binds NCOA4 and disrupts the NCO4-ferritin heavy chain 1 (FTH1) complex, causing the inhibition of ferritinophagy. Approaches that inhibit ferritinophagic regulators or stabilize ferritin complexes have shown potential in preclinical studies. However, the challenge lies in selectively modulating autophagy-related pathways without impairing their essential roles in cellular homeostasis [15,33,36].

Therapeutic targeting of the BECN1–SLC7A11 axis has shown encouraging preclinical results. Both genetic and pharmacological interventions have effectively modulated ferroptotic responses, suggesting potential applications in treating cancers and inflammatory conditions [18,32]. Nonetheless, due to the complexity of gastrointestinal environments, further investigation is needed to prevent off-target effects and unintended consequences [32].

**Figure 6:**
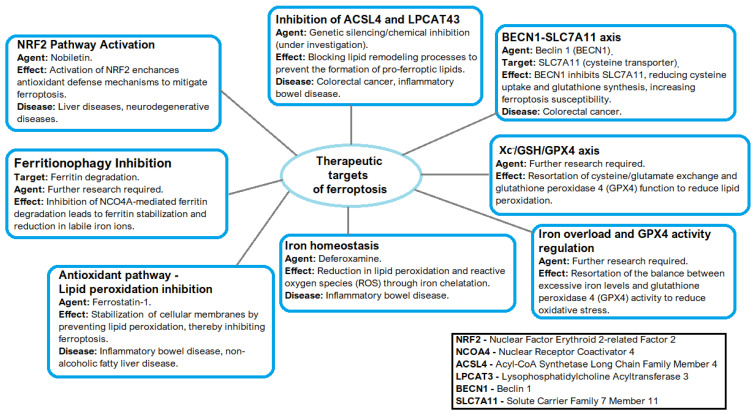
Therapeutics targets of ferroptosis.

The intricate interplay between iron metabolism and the Xc^−^/GSH/GPX4 axis adds complexity to ferroptosis regulation. Excess iron impairs GPX4 activity directly, posing challenges for therapeutic design [5]. As such, effective treatment strategies must simultaneously address iron levels and bolster antioxidant defenses. Striking this balance is critical for the development of targeted therapies for ferroptosis-associated gastrointestinal diseases [58]. Figure 6 summarizes the therapeutics targets of ferroptosis regulation discussed in this manuscript.

## 5. Clinical Applications and Future Directions

The clinical use of ferroptosis modulation represents a promising frontier for treating gastrointestinal diseases through targeted therapeutic strategies [5,51]. Ferroptosis inhibitors, such as Ferrostatin-1 and liproxstatin-1, have demonstrated efficacy in preclinical studies by suppressing lipid peroxidation, a central driver of ferroptosis. For example, Ferrostatin-1 has been shown to restore intestinal barrier integrity and reduce inflammation in colitis models, while liproxstatin-1 mitigates hepatocyte damage in alcoholic liver disease [61]. These compounds exert their effects by targeting enzymatic pathways, particularly GPX4, which plays a key role in regulating oxidative stress [18,51,61]. Despite their therapeutic potential, several challenges hinder clinical translation. These include limited pharmacokinetic properties and the risk of off-target effects. Optimizing bioavailability and assessing long-term safety are crucial steps toward their clinical use [62]. Additionally, combining ferroptosis inhibitors with anti-inflammatory agents may enhance therapeutic efficacy and offer a multifaceted approach to treating complex gastrointestinal conditions. Future studies exploring such synergistic combinations could expand clinical applications and improve patient outcomes [14,62].

Targeting GPX4 has emerged as a focused and versatile approach in managing ferroptosis-related gastrointestinal diseases. GPX4 protects against lipid peroxide-induced membrane damage and oxidative stress. Its inhibition, using compounds like RSL3, has been investigated as a strategy to selectively induce ferroptosis in cancer cells, including those in colorectal cancer and hepatocellular carcinoma [63]. Conversely, the activation of GPX4 has been shown to alleviate inflammation, enhance intestinal barrier function, and reduce oxidative stress in inflammatory bowel disease. This dual therapeutic potential—depending on the pathological context—highlights the importance of developing modulators with high specificity and low toxicity. Combining GPX4-targeted therapies with conventional treatments, such as chemotherapy or immunotherapy, has shown promise [64]. For example, the combination of RSL3 with imatinib has enhanced antitumor efficacy in drug-resistant gastrointestinal tumors [65].

In HCC, the co-administration of ferroptosis inhibitors with agents such as metformin has shown potential in reducing inflammation and restoring barrier function [66]. This multi-targeted therapeutic approach addresses the complexity of gastrointestinal pathologies while reducing the likelihood of resistance to monotherapy. However, further investigation is necessary to assess the safety, pharmacodynamics, and pharmacokinetics of these combinations, and to evaluate their efficacy across diverse patient populations [21]. Preclinical and clinical trials remain essential for validating their integration into clinical practice [26].

The gut microbiota also plays a pivotal role in ferroptosis regulation and its impact on gastrointestinal disease [67]. Certain microbial species contribute to disease progression by modulating ferroptotic pathways—for example, Peptostreptococcus anaerobius promotes colorectal cancer progression by producing metabolites that suppress ferroptosis. In contrast, probiotics can counteract these effects by enhancing antioxidant defenses and reducing lipid peroxidation [43,68]. These findings underscore the dual role of the gut microbiome and highlight the therapeutic potential of microbiota-based interventions, such as tailored probiotics or fecal microbiota transplantation [68]. However, the complexity of microbial ecosystems requires cautious approaches to avoid unintended dysbiosis [69].

Iron-chelating agents, including deferoxamine, have attracted attention for their ability to reduce ferroptosis-induced tissue injury by limiting the labile iron pool and decreasing ROS via inhibition of the Fenton reaction [70]. These agents have shown therapeutic benefits in diseases such as NASH and severe colitis, where iron overload contributes to oxidative stress. In cancer, iron chelation has been used to sensitize tumor cells to ferroptosis-inducing therapies, improving antitumor efficacy [71]. However, systemic iron chelation may lead to side effects such as anemia, necessitating the development of targeted delivery systems that limit action to pathological tissues. Combining iron chelation with other ferroptosis-modulating approaches, such as GPX4 inhibition, may enhance therapeutic precision and address the multifactorial nature of diseases like NASH and colorectal cancer [71]. Research should focus on optimizing these strategies to achieve a balance between safety and efficacy, especially in chronic-disease settings [5].

Advanced diagnostic tools are essential for tracking ferroptosis and assessing its role in disease. Techniques such as in situ imaging allow for the real-time detection of biomarkers like lipid peroxidation and GPX4 activity [23]. These tools enhance the ability to monitor treatment responses and detect diseases at earlier stages [62]. Integrating such diagnostics with therapeutic systems could pave the way for personalized medicine. For instance, hybrid platforms that combine ferroptosis monitoring with localized drug delivery may improve precision while simultaneously tracking disease progression [62]. Additionally, non-invasive diagnostics—such as blood-based biomarkers of lipid peroxidation—could revolutionize early intervention strategies [62,72]. Despite their promise, these technologies face hurdles related to cost and accessibility. Future efforts should aim to standardize methodologies and reduce expenses to facilitate their widespread clinical adoption [72].

## 6. Conclusions

Ferroptosis represents both a challenge and an opportunity in understanding and treating gastrointestinal diseases. This paper has reaffirmed its central role in driving pathology through iron metabolism dysregulation, oxidative stress, and lipid peroxidation. By elucidating the regulatory pathways—particularly the Xc^−^/GSH/GPX4 axis—and the involvement of genetic and metabolic factors such as Nrf2, ACSL4, and ferritinophagy, this study has clarified the mechanisms through which ferroptosis contributes to inflammatory, metabolic, and neoplastic conditions in the gastrointestinal tract.

The findings of this study underscore ferroptosis as a pivotal factor in epithelial damage in IBD, hepatocyte injury in liver diseases like NASH and ALD, and the complex tumor dynamics of colorectal cancer and HCC. Ferroptosis thus emerges as a promising therapeutic target, capable of being modulated contextually to either prevent tissue damage or enhance cancer cell death. While preclinical data provide compelling evidence, further clinical validation is essential to establish safety, efficacy, and reliable biomarkers for diagnosis and treatment monitoring.

Future research should aim to deepen our understanding of tissue-specific ferroptotic responses, investigate combinatory therapies—including immunomodulation and microbiota-targeted approaches—and leverage precision medicine to address individual susceptibilities. Advancing non-invasive detection tools and translational models will be critical in bridging bench-to-bedside gaps. Targeting this unique form of cell death may open new avenues to alleviating chronic inflammation, preventing organ failure, and overcoming cancer resistance.

Ultimately, ferroptosis research offers significant potential to transform clinical approaches in gastroenterology.

## Data Availability

Not applicable.
